# Scalp Micropigmentation Is an Effective Treatment for Localized Alopecia: Technical Analysis and a Series of Ten Case Reports

**DOI:** 10.1111/jocd.70375

**Published:** 2025-09-03

**Authors:** Qinsi Liu, Mang Sun, Jiezhi Zhang, Hengguang Zhao

**Affiliations:** ^1^ Department of Dermatology & Cosmetic Medicine Center The Second Affiliated Hospital of Chongqing Medical University Chongqing China

**Keywords:** cosmetic camouflage, localized alopecia, scalp micropigmentation, treatment protocol

## Abstract

**Background:**

Scalp micropigmentation (SMP) is emerging for camouflaging localized alopecia, yet standardized protocols and long‐term efficacy data remain limited.

**Aims:**

To evaluate technical parameters, clinical outcomes, and safety of SMP in diverse alopecia subtypes.

**Methods:**

Ten patients (androgenetic alopecia: *n* = 6; scarring alopecia: *n* = 4) underwent a standardized three‐session SMP protocol. Technical refinements included zone‐specific needle selection (single/triple point), hierarchical pigment deposition (prioritizing thick‐haired regions), and randomized distribution to avoid uniformity. Pigment density was incrementally adjusted (30%→70%→100% of natural follicular spacing). Adherence to a zero‐bleeding protocol ensured epidermal–upper dermal depth. Outcomes were assessed via visual density score (VDS, 0~10) and patient satisfaction score (PSS, 0~3).

**Results:**

All patients achieved significant cosmetic improvement. Immediate posttreatment VDS averaged 8.7 ± 1.1, with androgenetic cases scoring higher (9.1 ± 0.5). At 6‐month follow‐up, VDS declined modestly (7.7 ± 1.4), though scarring alopecia showed greater fading (Δ = 1.6 vs. Δ = 0.9 in androgenetic, *p* = 0.03). Patient satisfaction was high (mean PSS = 2.7/3), with 85.7% of androgenetic cases “very satisfied.” Strong correlation existed between VDS and PSS (*ρ* = 0.91, *p* < 0.001). No adverse events occurred.

**Conclusions:**

SMP is a safe, minimally invasive solution for localized alopecia, providing sustained cosmetic improvement. Technical refinements in needle selection, pigment layering, and depth control optimize outcomes. Future studies should focus on long‐term pigment retention and expanded cohorts.

## 1 Introduction

Tattoos are broadly classified into three categories based on their purpose and procedure: artistic, cosmetic, and medical. Artistic tattoos are primarily used for self‐expression and individuality [1], whereas cosmetic tattoos encompass semipermanent makeup applications such as eyeliner and eyebrows [2]. Medical tattoos refer to tattoos that serve a medical or therapeutic purpose. A significant advancement in the medical application of tattoos is scalp micropigmentation (SMP), which is increasingly recognized as an effective intervention for camouflaging hair thinning or loss [3, 4]. Unlike methods that attempt to increase real hair density, SMP employs tattoo techniques to simulate the appearance of cut hair shafts, thereby reducing scalp visibility [4, 5]. SMP's broad applicability to various alopecia types and its use in conjunction with hair transplantation enhance cosmetic outcomes [6].

In this study, we provide a detailed overview of the technical analysis, treatment intervals, and clinical outcomes of SMP in a cohort of several patients with localized alopecia. Through this exploration, we aim to enhance understanding of SMP's efficacy and broaden its acceptance as a treatment modality within the field of medical dermatology.

## 2 Case Presentations

Inclusion criteria is as follows: (1) stable condition of alopecia with no progression for at least 6 months; (2) absence of infectious inflammatory signs (e.g., redness, swelling, and purulent discharge) in the scalp; (3) no use of blood‐activating, stasis‐removing, enhancing microcirculation, or anticoagulant drugs within 1 week before SMP [6]; and (4) discontinuation of minoxidil treatment for at least 1 week before SMP.

The study was approved by the Ethics Committee of the Second Affiliated Hospital of Chongqing Medical University (Approval No. 2025‐LS‐133), and written informed consent was obtained from all participants prior to enrollment. The treatment protocol for all patients comprised three sessions administered at 1‐week intervals. During the initial session, pigment dot density was calibrated to 40 dots per square centimeter (dots/cm^2^). In the second session, the density was increased to 60 dots/cm^2^ to enhance coverage. By the final session, the density was further adjusted to 80–100 dots/cm^2^, achieving optimal naturalistic replication of follicular distribution and effective camouflage of alopecic areas.

**FIGURE 1:**
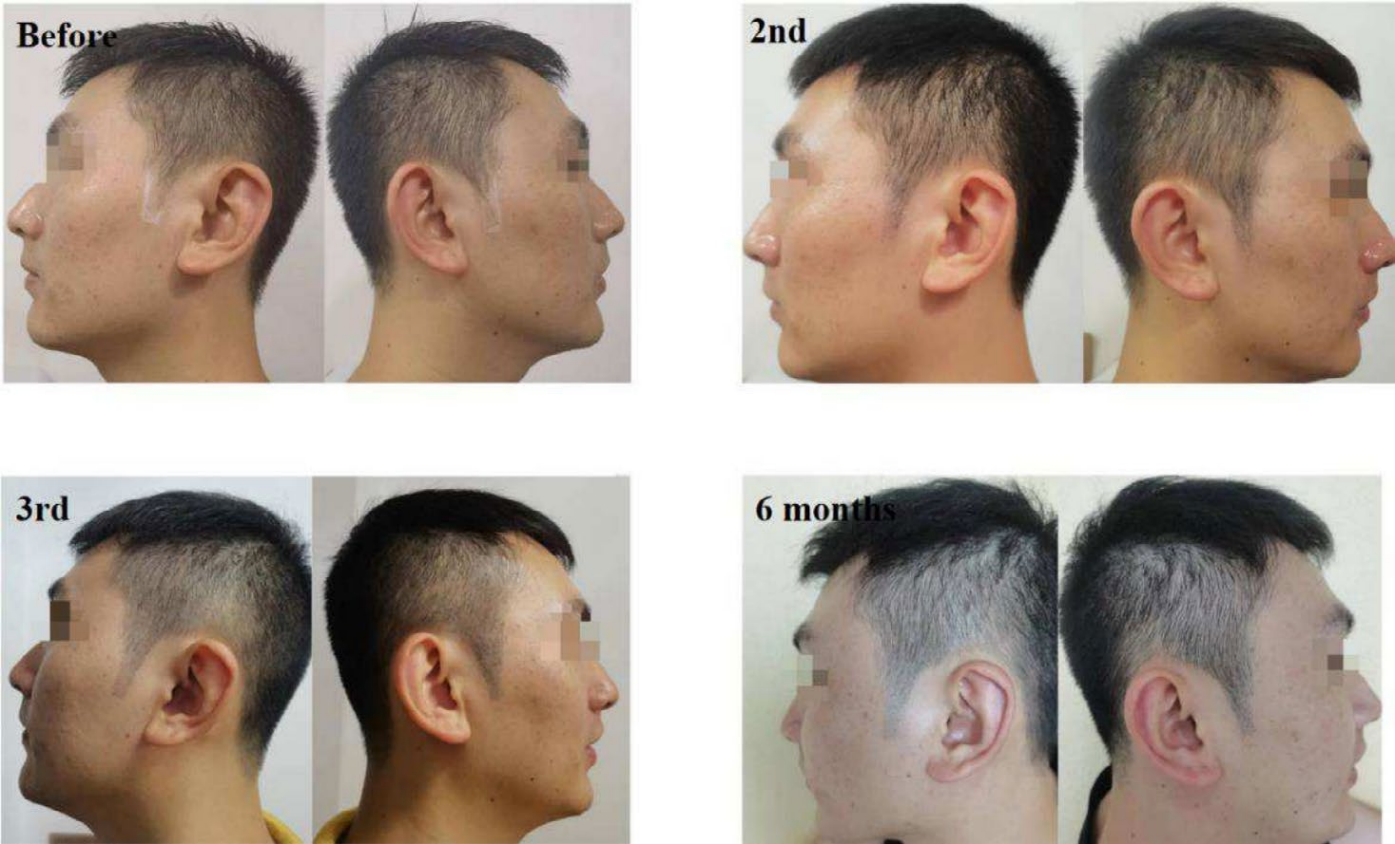
Thirty‐six‐year‐old Asian man with thinning hair on both temporal regions due to androgenetic alopecia and the condition of his bilateral temples before SMP treatment, post‐second session, post‐third session, and at the 6‐month follow‐up.

**FIGURE 2:**
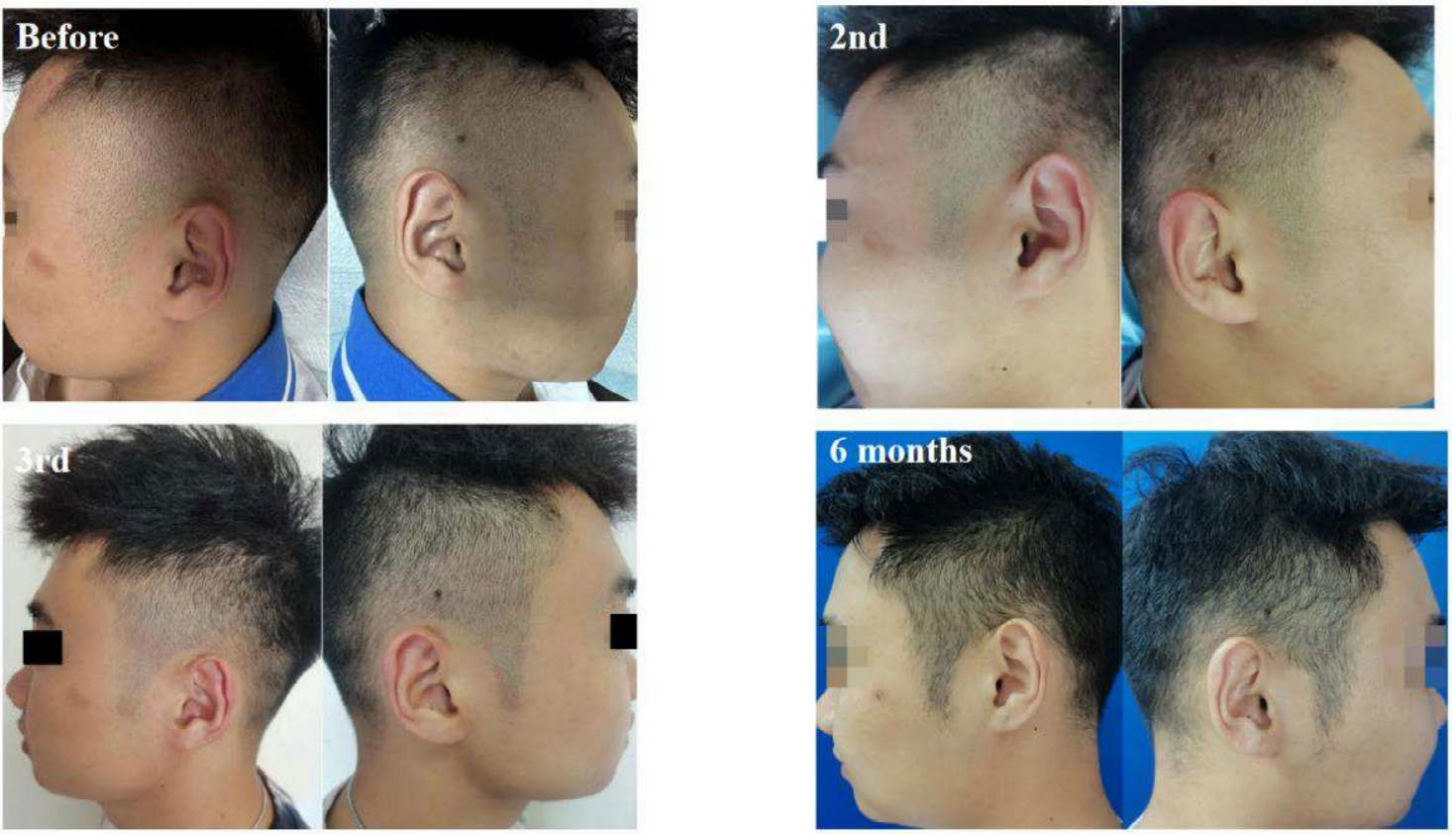
Twenty‐seven‐year‐old Asian man with thinning hair on both temporal regions due to androgenetic alopecia and the condition of his bilateral temples before SMP treatment, post‐second session, post‐third session, and at the 6‐month follow‐up.

Case 1 (Figure 1) and Case 2 (Figure 2) involve two male patients with androgenetic alopecia, each exhibiting bilateral temporal hair thinning unresponsive to pharmacological treatment. Both underwent SMP to augment hair density in these regions. Case 1 utilized tattoo needle models 0601RL (Round Liner), 0603RL, and 0803RL, while Case 2 used models 0601RL (Round Liner), 0801RL, and 0803RL.

**FIGURE 3:**
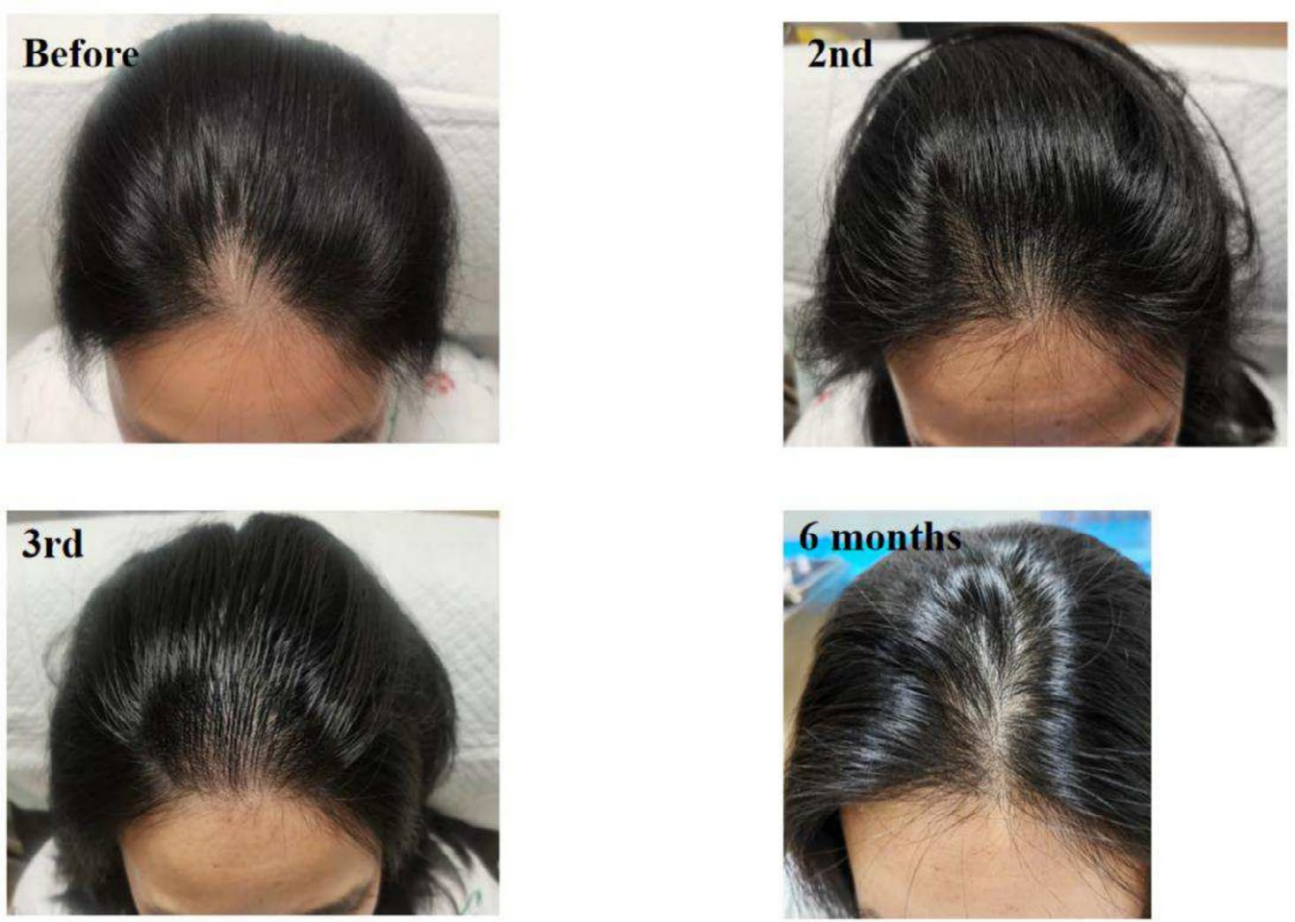
Thirty‐seven‐year‐old female patient with scarring alopecia at the frontal hairline following trauma. Images are shown before SMP treatment, after the second treatment, after the third treatment, and at a 6‐month follow‐up.

**FIGURE 4:**
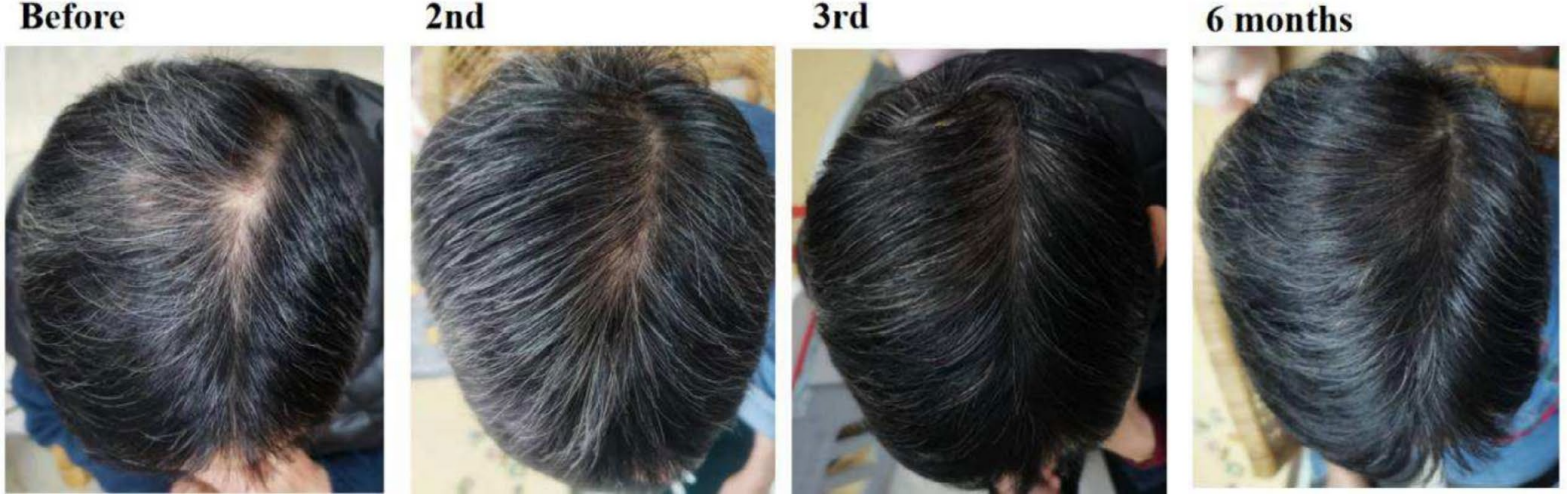
Seventy‐six‐year‐old female patient who developed scarring alopecia on the vertex of the scalp following trauma. The images presented include pre‐SMP treatment, post‐second treatment, post‐third treatment, and at a 6‐month follow‐up.

**FIGURE 5:**
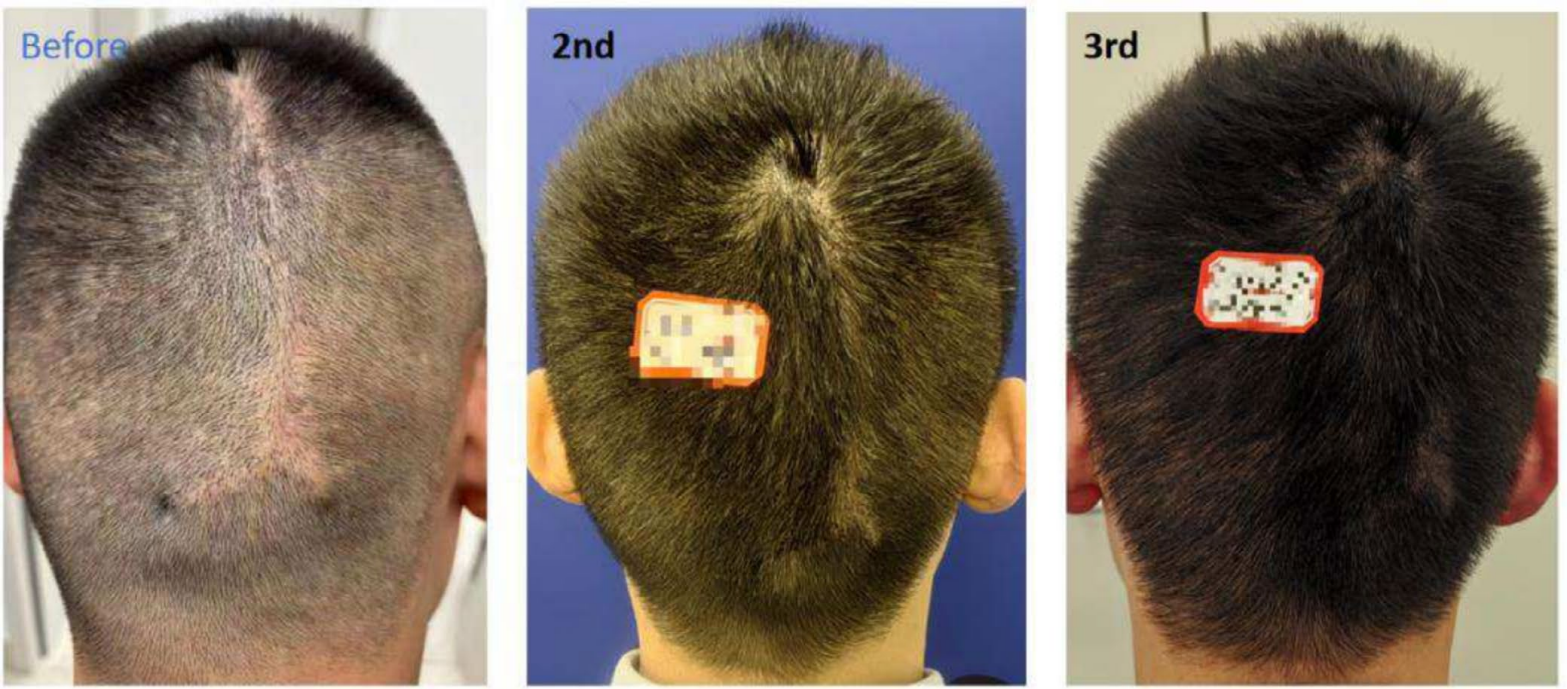
Thirty‐nine‐year‐old male patient with posttraumatic scarring alopecia. The images demonstrate the improvement in alopecia appearance before scalp micropigmentation (SMP) treatment, after two sessions, and after three sessions of SMP.

**FIGURE 6:**
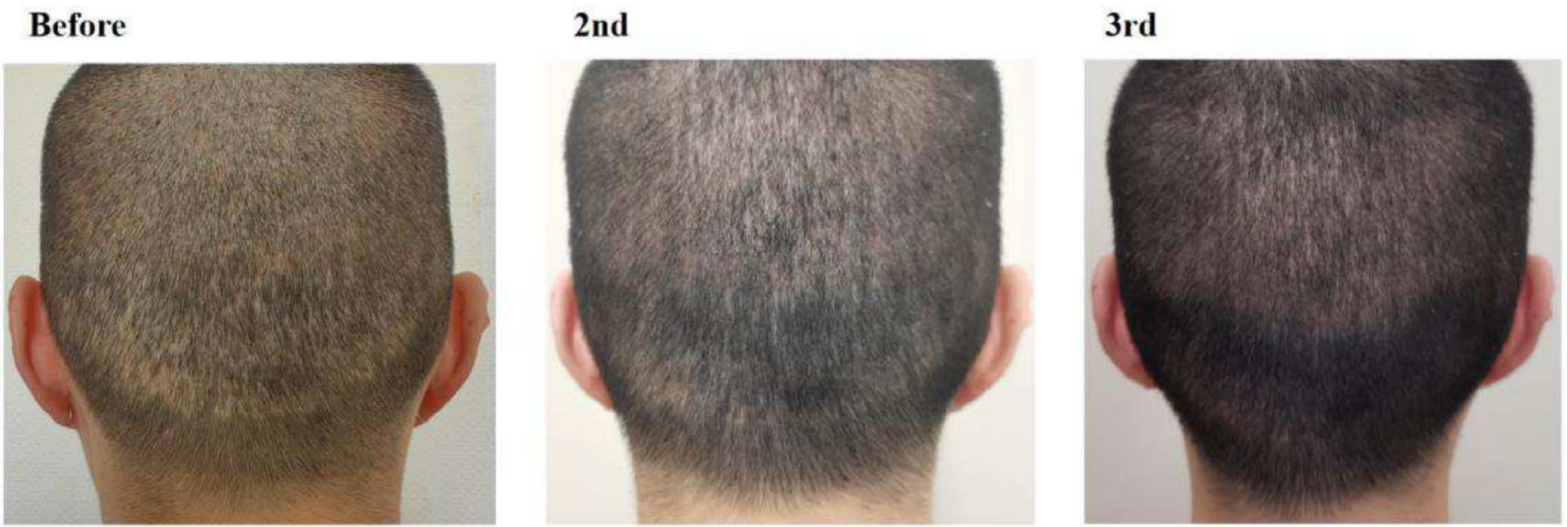
Thirty‐six‐year‐old male who developed moth‐eaten alopecia in the donor occipital area following hair transplantation. The images illustrate his condition before SMP treatment, after the second session, and following the third session.

Cases 3–6 (Figures 3, 4, 5, 6) detail four patients with scarring alopecia who underwent SMP to improve the appearance of hair loss. The first three cases involved scalp scars from trauma, while the last case involved occipital donor area scarring after hair transplantation. Case 4 (Figure 4) describes a 76‐year‐old female patient with type 2 diabetes, hypertension, and coronary artery disease, who was intolerant of traditional medications and surgical interventions but achieved satisfactory cosmetic improvements with SMP alone. Case 5 (Figure 5) had undergone two hair transplant surgeries at the scar site with minimal hair survival and ultimately opted for SMP. Case 3 (Figure 3) utilized tattoo needle models 0601RL (Round Liner), 0801RL, and 0803RL, while the latter three cases used models 0803RL and 1003RL.

**FIGURE 7:**
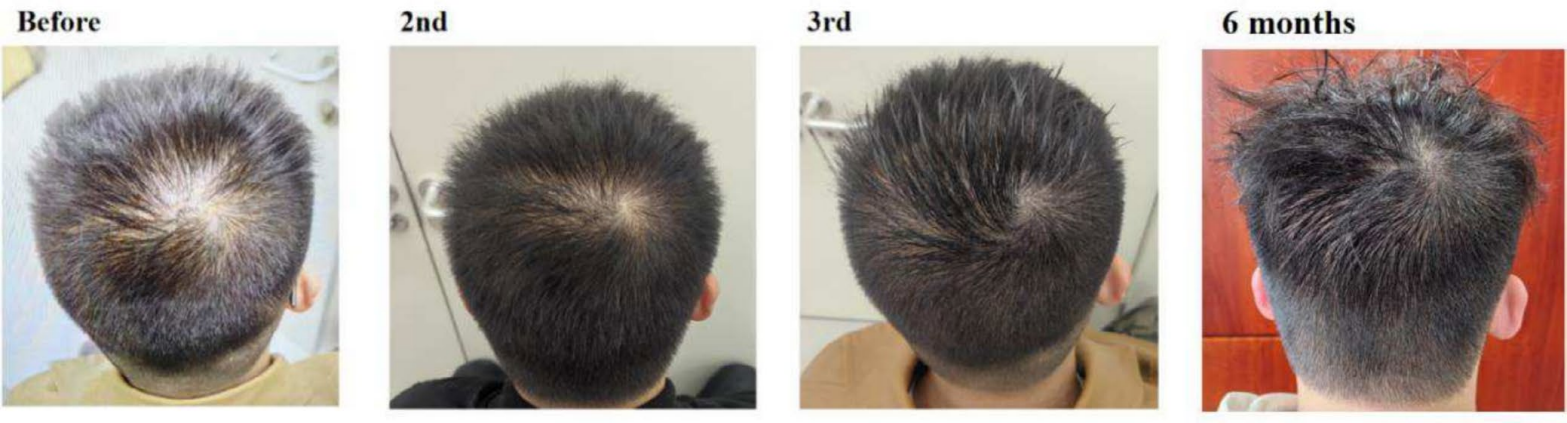
Sixteen‐year‐old male patient with androgenetic alopecia, primarily presenting as alopecia near the vertex area. The images display his condition before SMP treatment, after the second session, after the third session, and at the 6‐month follow‐up.

**FIGURE 8:**
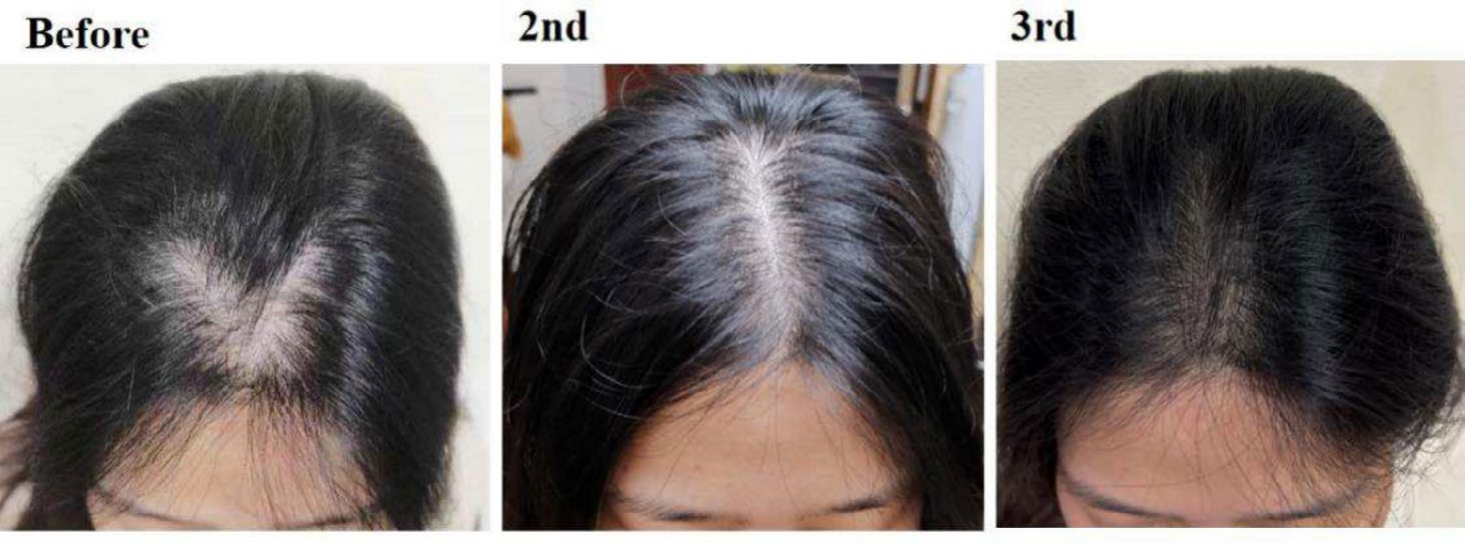
Twenty‐four‐year‐old female patient with androgenetic alopecia, primarily manifesting as alopecia on the scalp vertex. The images show her condition before SMP treatment, after the second session, and after the third session.

**FIGURE 9:**
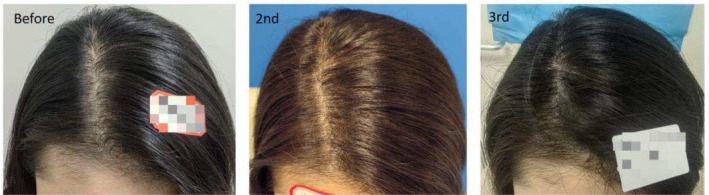
Thirty‐eight‐year‐old female patient with a widened parting in the vertex area caused by androgenic alopecia. The images demonstrate the appearance of the parting before scalp micropigmentation (SMP) treatment, after two sessions, and after three sessions of SMP.

**FIGURE 10:**
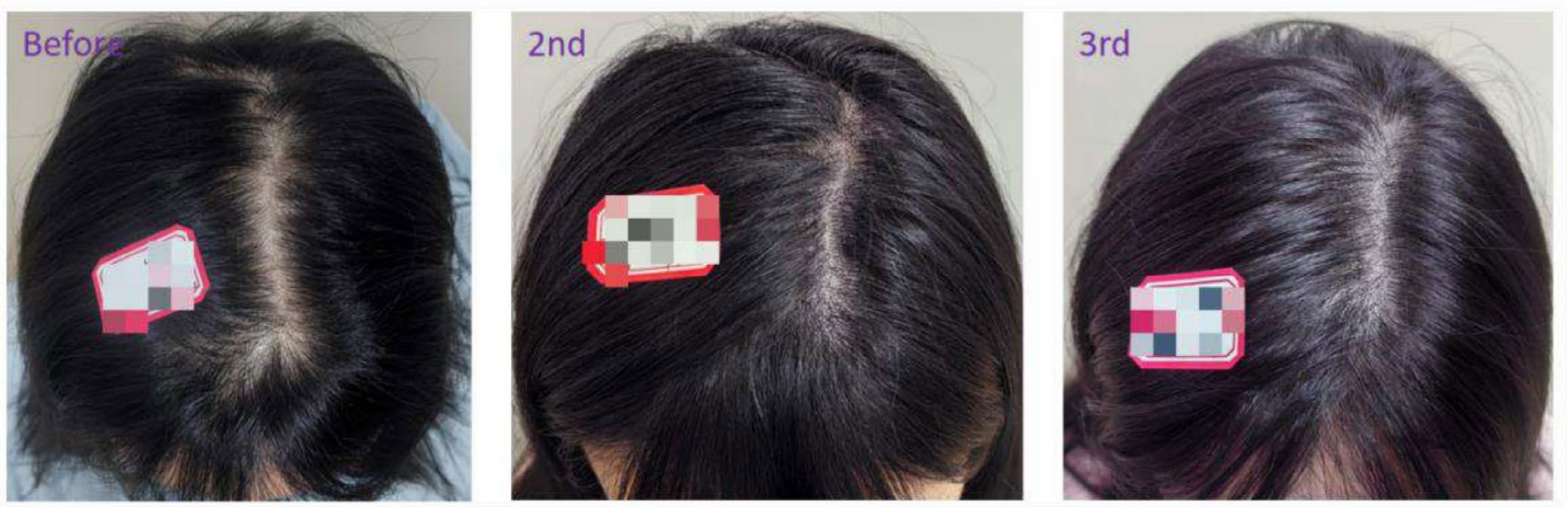
Thirty‐two‐year‐old female patient with androgenic alopecia presenting as widened vertex parting. The images illustrate the improvement in parting appearance prior to scalp micropigmentation (SMP), after two sessions, and following three sessions of SMP.

**FIGURE 11:**
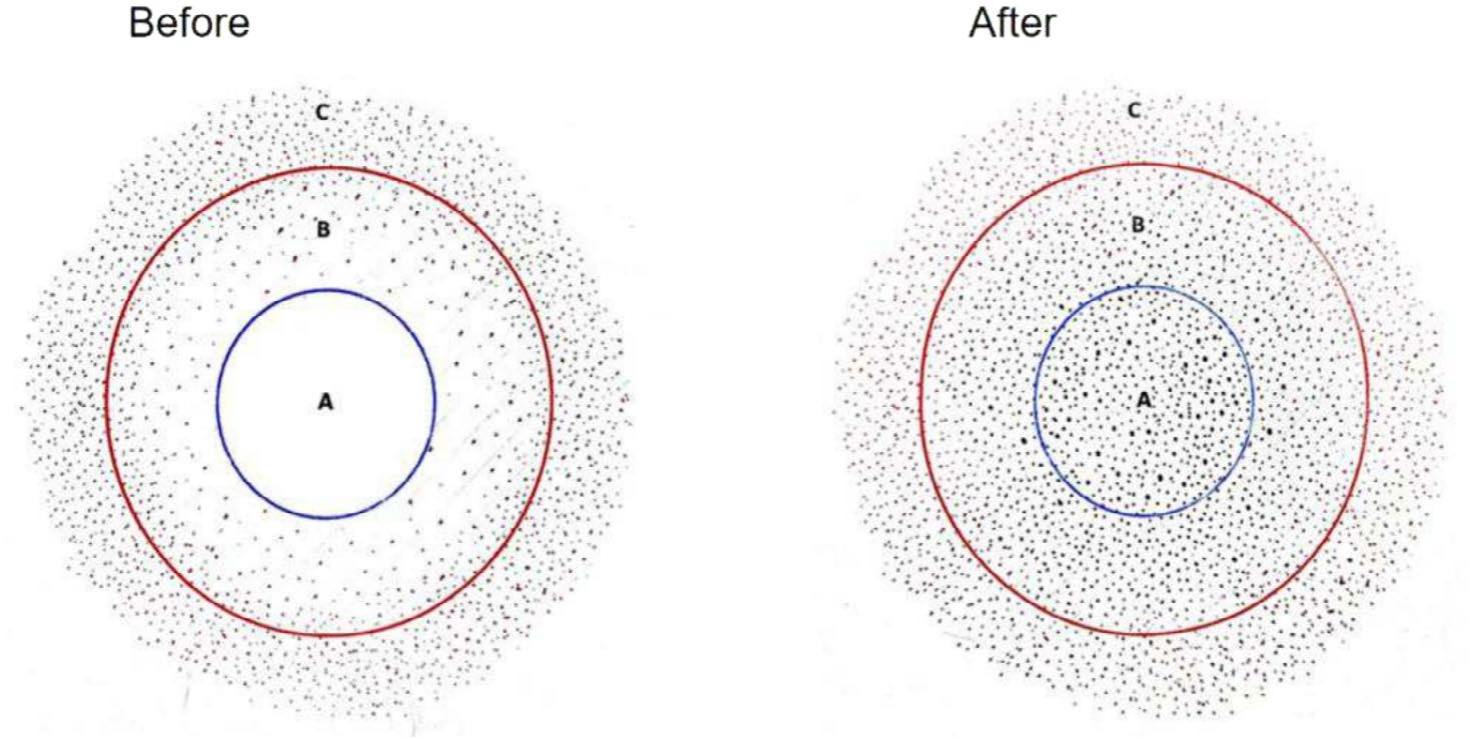
The left image illustrates pre‐SMP hair distribution: Area A (complete hair loss), Area B (thinning hair), and Area C (normal density). These areas should be marked prior to SMP with blue and red circles, as shown. The right image depicts recommended post‐SMP pigment placement: Area A should achieve normal hair density, while Area B should have progressively reduced pigment density for a seamless transition to Area C.

**FIGURE 12:**
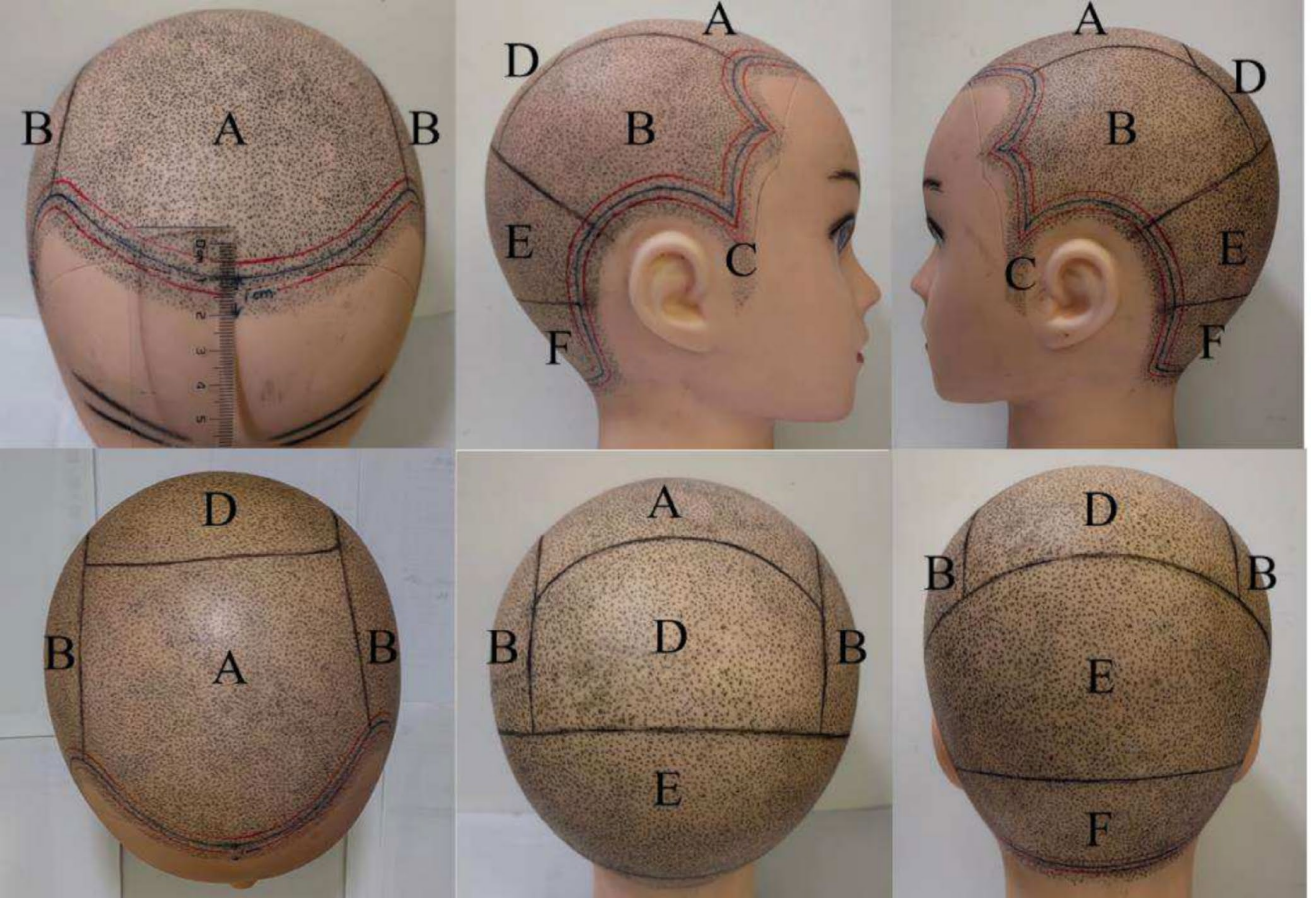
A 1‐cm zone is delineated extending inward from the leading edge of the hairlines and the neck area, as indicated by the blue line in the figure. A single‐pronged needle with a 0.2 mm diameter is advised for use within these hair borders. For the vertex (A), temporal region (B), whorl area (D), occipital region (E), and nuchal area (F), a triple‐pronged needle with a 0.25 mm diameter is recommended. To assist in the transition between these regions, a single‐pronged needle with a 0.25 mm diameter may be utilized, as indicated between the red lines. The recommended sequence for pigment deposition prioritizes areas with thicker hair shafts (e.g., zones D, A, E, F, and B as illustrated), followed by finer‐haired regions (e.g., the hairlines and the neck area).

**FIGURE 13:**
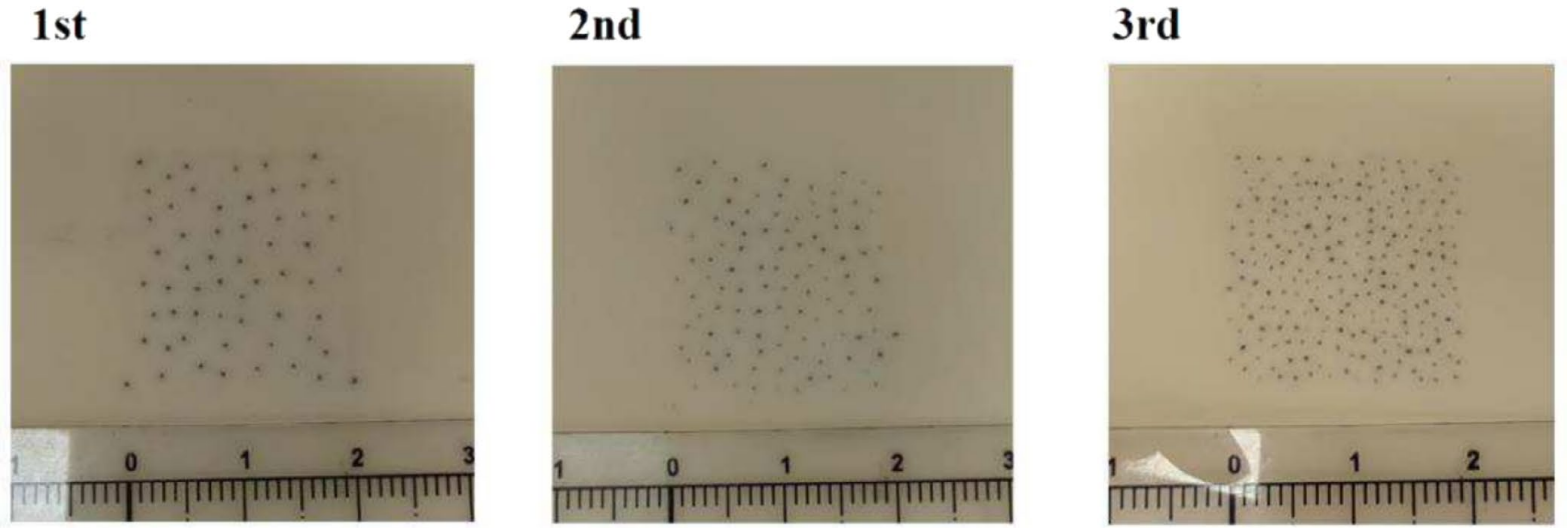
Pigment density progression: 40 dots/cm^2^ (initial), 60 dots/cm^2^ (second), 80–100 dots/cm^2^ (final).

**FIGURE 14:**
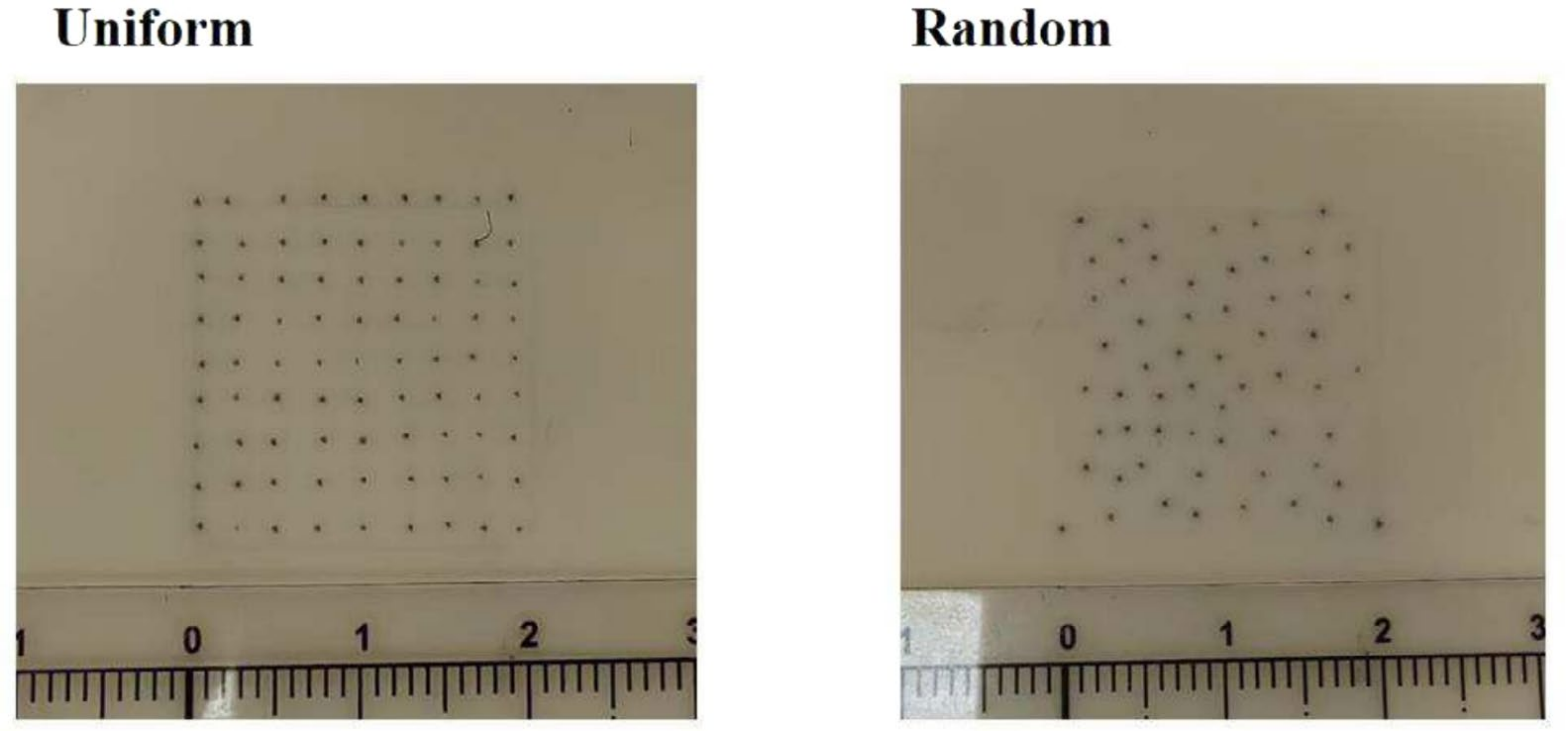
Pigment placement should adhere to the principle of random distribution (Figure 14, right), as a uniform pattern (Figure 14, left) can result in an unnatural appearance.

Cases 7–10 (Figures 7, 8, 9, 10) present four patients with androgenetic alopecia who underwent SMP for cosmetic enhancement of hair loss appearance. Case 7 was an adolescent male unable to tolerate long‐term traditional medication, who received SMP to improve the appearance of vertex hair loss. Case 8 received SMP to address frontal hairline thinning. Cases 9 and 10 involved female patients with diffuse thinning on the crown, who experienced inadequate results from over a year of traditional treatments including spironolactone and minoxidil, and opted for SMP to improve part line density. Case 7 used tattoo needle models 0803RL and 1003RL, while cases 8–10 used models 0601RL, 0801RL, and 0803RL.

## 3 Results

The outcomes were evaluated using two validated scoring systems: (1) visual density score (VDS, 0~10), assessing pigment coverage and naturalness of hair simulation, and (2) patient satisfaction score (PSS, 0~3), capturing subjective perceptions of esthetic improvement (Tables 1 and 2). SMP demonstrated relatively long‐term significant cosmetic improvement in localized alopecia, with immediate posttreatment VDSs averaging 8.7 ± 1.1 (range: 6.1~9.8), where androgenetic alopecia cases achieved higher scores (mean: 9.1 ± 0.5), and 85.7% of androgenetic alopecia patients (6/7) reported “very satisfied” outcomes (score = 3) (Table 1). At 6‐month follow‐up (cases 1~4 and 7), a mean reduction of 1.0 ± 0.5 points in visual density (from 8.7 ± 1.1 to 7.7 ± 1.4) was observed, with scarring alopecia (case 3) showing greater decline compared to androgenetic cases (Δ = 1.6 vs. Δ = 0.9, *p* = 0.03) (Table 2). All androgenetic alopecia patients (Cases 1, 2, and 7) and Case 4 maintained “very satisfied” ratings, underscoring SMP's durability in nonscarred regions (Table 2). Statistical analysis revealed a strong correlation between visual density and patient satisfaction (Spearman's *ρ* = 0.91, *p* < 0.001), with androgenetic alopecia exhibiting superior density retention (Δ = 0.9 vs. Δ = 1.6, *p* = 0.04) (Tables 1 and 2). These findings validate SMP as a rapid, minimally invasive solution for localized alopecia, including challenging scarring cases, achieving high patient satisfaction (mean: 2.7/3) and sustained cosmetic outcomes (Tables 1 and 2).

**TABLE 1:** Immediate posttreatment.

Case	Diagnosis	Visual density score (0–10)^a^	Patient satisfaction (0–3)^b^
1	Androgenetic alopecia	9.5 ± 0.5	3
2	Androgenetic alopecia	9.8 ± 0.2	3
3	Scarring alopecia	6.1 ± 0.5	2
4	Scarring alopecia	9.2 ± 0.3	3
5	Scarring alopecia	6.3 ± 0.7	2
6	Scarring alopecia	8.1 ± 0.9	3
7	Androgenetic alopecia	8.7 ± 0.3	3
8	Androgenetic alopecia	9.6 ± 0.4	3
9	Androgenetic alopecia	7.5 ± 0.5	2
10	Androgenetic alopecia	8.9 ± 0.1	3

^a^ Visual density score (0–10): 0: no improvement, 3: mild improvement (< 50% pigment coverage, blurred boundaries), 6: moderate improvement (50%–80% coverage, natural gradient), 10: significant improvement (> 80% coverage, indistinguishable from natural hair).

^b^ Patient satisfaction score (0–3): 0: dissatisfied (no improvement or worsening), 1: neutral (minor improvement, below expectations), 2: satisfied (marked improvement, met expectations), 3: very satisfied (exceeded expectations, enhanced social confidence).

**TABLE 2:** Six‐month follow‐up (Cases 1–4 and 7).

Case	Diagnosis	Visual density score (0–10)^a^	Patient satisfaction (0–3)^b^
1	Androgenetic alopecia	8.8 ± 0.2	3
2	Androgenetic alopecia	8.9 ± 0.1	3
3	Scarring alopecia	5.5 ± 0.5	1
4	Scarring alopecia	8.9 ± 0.2	3
7	Androgenetic alopecia	7.8 ± 0.2	3

^a^ Visual density score (0–10): 0: no improvement, 3: mild improvement (< 50% pigment coverage, blurred boundaries), 6: moderate improvement (50%–80% coverage, natural gradient), 10: significant improvement (> 80% coverage, indistinguishable from natural hair).

^b^ Patient satisfaction score (0–3): 0: dissatisfied (no improvement or worsening), 1: neutral (minor improvement, below expectations). 2: satisfied (marked improvement, met expectations). 3: very satisfied (exceeded expectations, enhanced social confidence).

## 4 Technical Analysis

The standard operating procedure for SMP involves several precise steps to ensure successful outcomes [3, 7, 8]. Initially, define the overall area designated for SMP by mapping it into zones of hair loss and transitional areas (Figure 11). Special attention should be paid to areas such as the anterior hairline and the posterior hairline margin marking a boundary that typically extends 1 cm inward from these lines (Figure 12). This preparatory step ensures proper planning for pigment application. The subsequent step involves the meticulous selection of needle sizes, specifically adapted to correspond with the patient's inherent hair fiber thickness. In general, finer hair is observed along the anterior hairline and the posterior hairline margin, where a 0.2‐mm single‐point needle is recommended (Figure 12) [7, 8]. In contrast, the rest of the scalp exhibits uniformly dense hair, for which a three‐point needle with a diameter of at least 0.25 mm is advised (Figure 12) [7]. For transitional boundary areas between these zones, a 0.25‐mm single‐point needle may be used to achieve a seamless transition (Figure 12). Following the design established in the first two steps, pigment is deposited using the selected needle sizes in regional sections. The procedure begins from central locations and moves outward, systematically progressing from areas with coarser hair to finer hair diameters (Figure 12). Notably, pigment application at the hairline demands heightened caution, as ink diffusion in this area may lead to perceptible blurring and requires meticulous technique to avoid detectable artifacts.

Generally, particularly in cases of extensive hair loss, SMP is a gradual process that typically requires 3–5 treatment sessions spaced 5–7 days apart to progressively increase pigment density [6, 8, 9]. During the initial session, pigment dot density is calibrated to 40 dots per square centimeter (dots/cm^2^). In the second session, the density is increased to 60 dots/cm^2^. By the final session, the density is further adjusted to 80–100 dots/cm^2^ to achieve natural‐looking coverage that effectively camouflages alopecic areas (Figure 13) [6]. In contrast, for small‐area scar alopecia, multiple sessions are not always necessary, and a single treatment may suffice to achieve the desired density and esthetic outcome [6]. Several weeks following the completion of targeted SMP, an overall assessment should be conducted to evaluate pigment retention, as immune cells may clear the pigment over time [8, 10]. The pigment should mimic the appearance of cut hair shafts in both thickness and density, demonstrating randomness, uniformity, and gradient [10]. If certain areas do not meet these objectives, an additional 1–2 targeted sessions may be required for correction [6, 8, 9, 11].

Posttreatment, monitor the scalp for any coloring reactions and adjust treatment parameters as needed for subsequent sessions. Signs such as localized redness, exudation, or blistering may indicate an allergic reaction to the pigment, necessitating cessation of the procedure and application of topical corticosteroids until recovery [9]. In cases of pigment fading, the depth of pigment placement should be increased; conversely, blurring may require a reduction in placement depth [6, 7, 12]. The ideal depth for SMP placement lies above the dermal papillary layer and below the basal epidermal layer, which, given the variable thickness and undulating surface of the scalp epidermis, makes precise depth control both critical and challenging [3, 6, 7]. Therefore, adherence to a zero‐bleeding protocol is critical during the procedure. Hemorrhage signifies needle penetration into the dermal papillary layer with disruption of papillary capillaries, necessitating immediate reduction of pigment implantation depth [3, 7, 11]. Based on our clinical experience, blurring primarily results from applying excessive pigment within a localized area when adhering to the no‐bleeding protocol, necessitating reduced pigment volume. For pigment fading observed within 1 month post procedure, we recommend primary escalation of pigment dosage. If fading recurs within the same timeframe despite dosage adjustment, a cautious increase in implantation depth (≤ 0.1‐mm increments) may be implemented, while rigorously maintaining the zero‐bleeding protocol. This stepwise approach identifies the optimal pigment placement and dosage to balance effectiveness with safety. Pigment should be applied following a randomized distribution pattern to avoid unnatural uniformity (Figure 14), especially around the hairline, sideburns, or edges [7]. In transitional zones, from sparsely haired regions to normal‐density areas, pigment should be placed in the interfollicular spaces of the scalp, with density gradually decreasing to create a seamless blend with surrounding natural hair (Figure 11). Postpigment deposition, a bluish hue commonly manifests due to dermal light scattering, wherein shorter‐wavelength cool tones (e.g., blue spectrum) are preferentially reflected by superficial cutaneous layers, while long‐wavelength warm tones (e.g., red spectrum) are absorbed by deeper tissues [10]. Even when pure black pigment is used, a bluish chromatic shift occurs across all skin types (including African descent) due to epidermal light refraction [10]. The recommended solution involves creating microdots (diameter < 0.5 mm), which exploit human visual acuity limitations to blur the chromatic discrepancy between blue and black hues, achieving seamless optical fusion [10]. When performing SMP on scar tissue, increased skin resistance necessitates a higher rotor speed for effective pigment application [7]. Post‐SMP care requires avoidance of scalp washing and alcohol‐based disinfectants for 72 h due to persistent epidermal microchannels, which may induce pigment discoloration.

## 5 Discussion

Currently, there is a lack of effective pharmacological or surgical treatments for diffuse thinning hair and permanent alopecia. Hair transplantation is one of the main treatments, while Asian individuals face the challenges of flatter skulls, higher scalp tension, coarser hair follicles, and increased posttransplant scarring [3, 11]. Coupled with lower donor hair density compared to Caucasians, these factors raise the risk of donor site depletion, as similar graft volumes are necessary to cover recipient areas [11, 12]. Pinpoint scars from follicular unit extraction can restrict esthetic outcomes, such as achieving a dense “shaved head style,” and excessive extraction may result in transparent, moth‐eaten donor areas [12]. In this context, SMP is gaining recognition as a highly effective intervention for improving the appearance of diffuse thinning and scarring alopecia posttransplantation [13]. SMP offers advantages such as reduced visible donor area depletion, minimized surgical trauma, and alleviation of emotional and financial burdens [6, 12]. When combined with hair transplantation, SMP can facilitate the restoration of a full head of hair appearance, even in patients with severe balding patterns and limited donor supply [12].

The final appearance of SMP is closely related to several factors: the depth and size of the pigment insertion, the needle type selected for different scalp areas, the distribution design of pigment density (particularly in transitional areas), the number of treatment sessions, the gradual layering of pigment density, scalp thickness, resistance, scalp health condition, pigment color, and pigment stability [3, 11]. This study primarily evaluates the efficacy of SMP in patients with localized alopecia. Our findings demonstrate its marked effectiveness in individuals with thinning hair or limited alopecia areas who have not achieved their desired volumetric restoration [5, 6]. Notably, SMP exhibits broad applicability across diverse alopecia subtypes and severity levels, including alopecia totalis. When performed with proper technique and combined with tailored esthetic design, SMP achieves significant cosmetic improvement in hair loss appearance. Some researchers suggest that if reduced hair caliber, rather than decreased hair density, is the primary cause of scalp visibility, hair transplant surgery could potentially exacerbate the condition by damaging adjacent hair follicles [5]. In such cases, SMP may be a more appropriate option. We posit that these may be associated with the phenomenon of persistence of vision in human eyes. Currently, the depth of pigment placement in SMP relies solely on the clinician's tactile expertise, as no sensitive instrumentation exists for precise depth control [7]. Mastering this technique requires months of dedicated practice to develop the necessary tactile discrimination—a critical learning curve that underscores the procedure's technical demands and should not be underestimated. It continues to face challenges regarding the longevity and stability of its long‐term outcomes. If the pigment is inserted too deeply, it can diffuse over time, leading to an irreversible patchy appearance on the scalp, with the exact mechanism not yet fully understood [9, 10, 11]. Studies indicate that after the treatment conclusion, pigments are retained in dermal macrophages, which cannot degrade them [14]. These macrophages release pigments upon their death, with new macrophages continuously replacing them, thus sustaining the pigments in situ and ensuring the permanence of SMP [13]. All micropigmentation procedures exhibit gradual fading over time, even with metabolically inert pigments. Several mechanisms are hypothesized to contribute to this phenomenon: Partial fading may result from pigment migration to regional lymph nodes and systemic distribution, while enzymatic degradation also plays a role [8, 14]. Consequently, preprocedural counseling must emphasize the nonpermanent nature of SMP outcomes, particularly in patients with scarring alopecia, who may require touch‐up procedures within several years. Additionally, sunlight exposure may cause pigment fading or color change, necessitating patient adherence to protection against ultraviolet exposure [3]. However, the long‐term health implications of these processes, as well as the body's response to UV interaction with the pigments, remain insufficiently understood [3, 14].

Future developments might focus on precisely controlling pigment placement depth and enhancing SMP techniques to improve integration with existing hair, create more realistic appearances, and reduce the invasiveness of treatment. In‐depth research into the behavior of pigments within the body and their correlation with stability will further reduce unfavorable outcomes in the esthetic improvement of SMP. The case reports in this article are limited in both number and types of alopecia. The 6‐month follow‐up period in this study may be inadequate for detecting pigment fading, as these changes (particularly UV‐induced chromatic alterations) generally become clinically observable after 6 months. Extended follow‐up observations would be valuable to assess the long‐term efficacy and safety of SMP. Larger sample sizes, inclusion of more diverse alopecia types, and extended follow‐up studies are necessary to determine the enduring and stable effects of SMP on improving the appearance of alopecia.

## 6 Conclusions

SMP is not intended for diagnosing, preventing, and treating diseases. Similar to other tattooing tools and pigments, it serves as a cosmetic procedure aimed at concealing unsightly scars, deformities, baldness, and areas of thinning hair. Compared to conventional therapies, SMP is deemed safer due to its minimal and fewer side effects. In conclusion, for individuals experiencing localized alopecia, SMP presents itself as a rapid, effective, minimally invasive, cost‐efficient, and safe solution. The utilization of SMP could potentially offer healthcare professionals an opportunity to address this significant yet untapped market demand.

## Conflicts of Interest

The authors declare no conflicts of interest.

## Data Availability

The data that support the findings of this study are openly available in Mendeley Data at https://data.mendeley.com/datasets/8yp43vgkkj/1, reference number V1.
